# Lung Function in Preschool Children in Low and Middle Income Countries: An Under-Represented Potential Tool to Strengthen Child Health

**DOI:** 10.3389/fped.2022.908607

**Published:** 2022-06-06

**Authors:** Shaakira Chaya, Heather J. Zar, Diane M. Gray

**Affiliations:** Department of Paediatrics and Child Health, Red Cross War Memorial Children's Hospital and SA-MRC Unit on Child and Adolescent Health, University of Cape Town, Cape Town, South Africa

**Keywords:** spirometry, fractional exhaled nitric oxide, oscillometry, interrupter technique, tidal breathing, multiple breath washout

## Abstract

**Background:**

The burden of respiratory disease is high in low-middle income countries (LMIC). Pulmonary function tests are useful as an objective measure of lung health and to track progression. Spirometry is the commonest test, but its use is limited in preschool children. Other lung function methods have been developed but their use in LMIC has not been well described.

**Aim:**

To review the use of preschool lung function testing in children in LMIC, with particular reference to feasibility and clinical applications.

**Methods:**

Electronic databases “PubMed”, “Scopus”,” Web of Science”, and “EBSCO host” were searched for publications in low and middle income countries on preschool lung function testing, including spirometry, fractional exhaled nitric oxide (FeNO), oscillometry, interrupter technique, tidal breathing and multiple breath washout (MBW), from 1 January 2011 to 31 January 2022. Papers in English were included and those including only children ≥6 years were excluded.

**Result:**

A total of 61 papers from LMIC in Asia, South America, Africa, Eurasia or the Middle East were included. Of these, 40 included spirometry, 7 FeNO, 15 oscillometry, 2 interrupter technique, and 2 tidal breathing. The papers covered test feasibility (19/61), clinical application (46/61) or epidemiological studies (13/61). Lung function testing was successful in preschool children from LMIC. Spirometry was the most technically demanding and success gradually increased with age.

**Conclusion:**

Preschool lung function testing is under-represented in LMIC for the burden of respiratory disease. These tests have the potential to strengthen respiratory care in LMIC, however access needs to be improved.

## Introduction

Childhood respiratory disease is a common cause of morbidity and mortality globally (1). The burden of acute and chronic respiratory disease is especially high in low-middle income countries (LMIC) (2), may result in impaired lung function and set a trajectory for chronic illness into adulthood (3, 4). However, access to respiratory diagnostic and management tools such as lung function are limited in many LMIC (5).

Lung function attained in early life is important for respiratory health, with low lung function associated with subsequent risk of respiratory disease (6). Pulmonary function tests are an objective measure of lung health which can be used to diagnose and track lung disease and assess response to treatment. In recent years non-invasive tests have been developed and guidelines produced for preschool children, facilitating its use in assessing respiratory health in early life (7, 8).

Lung function tests used in preschool children include spirometry, bronchial response testing, multiple breath washout (MBW), fractional exhaled nitric oxide (FeNO), oscillometry and other tests which measure resistance, including the interrupter technique (Rint). and plethysmography. With increased recognition of the importance of maximizing early life respiratory health and the growing availability of tools to do so, their use in LMIC is of particular interest.

Of the preschool lung function tests, spirometry is the most commonly used and most widely available. Even though use is limited in very young or uncooperative children, it is feasible in children as young as 3 years (9). Spirometry measures lung volumes at maximal expiration and is able to assess airflow obstruction, response to bronchodilator therapy and lung volumes on forced expiration. The commonly reported measures are the forced expiratory volume in the first second (FEV_1_), forced vital capacity (FVC), FEV_1_/FVC and forced expiratory flow between 25 and 75% of FVC (FEF_25−75_). (10). Spirometry predominantly reflects airflow in large and medium sized airways, and is a poor measure of peripheral small airways or early lung disease (11). Current international recommendations for spirometry collection and interpretation in young children are available (10, 12).

Oscillometry (or the forced oscillation technique, FOT). is a simple, non-invasive technique which is performed during tidal breathing. Minimal co-operation is required thereby making this a popular measurement in preschool children. This measures the impedance of the respiratory system, which includes resistance and reactance across a range of frequencies reflecting the entire respiratory system including the small airways (13). The novel intra-breath measurement may be a more sensitive measure of small airway disease, thus allowing early detection of disease (14, 15). Recent international recommendations for oscillometry have been published on methodology, technical standards and future developments for use in children (7, 16, 17).

The interrupter technique measures the resistance of the respiratory system requiring minimal cooperation. The technique involves a sudden interruption of flow during tidal breathing, this allows for alveolar and pressure at the mouth to equilibrate therefore alveolar pressure can be estimated (18, 19). Different methods have been used to perform the test making the comparison of results difficult thus highlighting the need for test standardization. Furthermore, research is still required to determine the best algorithm to calculate pressure at the mouth during occlusion and the cut-off value for Rint post bronchodilator needs to be established (7).

Multiple breath washout (MBW) is used to assess ventilation homogeneity. It measures the functional residual capacity (FRC) and the lung clearance index (LCI) in preschool children (8). It is more sensitive than spirometry for the detection of peripheral airway disease and has been successfully used in the monitoring of children with cystic fibrosis (CF) and primary ciliary dyskinesia (PCD) (7, 20). It correlates with high resolution CT scan in CF patients as well as in children with asthma, bronchiolitis obliterans or chronic lung disease of prematurity (8, 21).

Fractional exhaled nitric oxide (FeNO) is a non-invasive marker of T-helper cell type 2 (TH2). eosinophilic airway inflammation. Tests can be performed with high repeatability and accuracy (22–24). Its main use is an adjunct to the diagnosis of TH2 type asthma and guiding the use of inhaled corticosteroid (ICS) treatment. In addition, FeNO can also assist in the differential diagnosis of other conditions such as cystic fibrosis (CF), PCD, scleroderma, obstructive sleep apnoea syndrome and hepatopulmonary syndrome. Fractional exhaled nitric oxide levels are low in PCD; however measurement of nasal NO improves the diagnostic accuracy and is a useful screening tool for PCD (23, 25). Normal values for FeNO are published for children from 4 years of age (24), as well as international applications and the use of FeNO (23, 24, 26). However, there are limited data on the use and feasibility of such measures in LMIC, despite the high burden of lung disease and need for objective tools to diagnose and monitor these.

We aimed to review the use of preschool lung function testing in children in LMIC, with reference to feasibility and clinical applications, to identify opportunities for optimizing diagnosis and management of childhood respiratory disease in these settings.

## Methods

We reviewed published literature of preschool lung function testing in LMIC, which included children between the ages of 3 to 5 years. We included published papers from 1 January 2011 to 31 January 2022 that included lung function testing in the preschool age group from a World Bank defined LMIC.

The search was conducted on the following electronic databases: PubMed, Scopus, EBSCOhost (Cinahl, Africa wide information, Health source- Nursing/Academic Edition). and Web of Science including the search terms: Respiratory Function Test^*^” OR “Lung function test^*^” OR “pulmonary function test^*^” OR “respiratory function test^*^” OR “multiple-breath washout” OR “Forced oscillation technique” OR” tidal breathing” OR “fractional excretion of nitric oxide” OR Spirometry OR Oscillometry OR “impulse oscillometry” OR “interrupter technique” OR “interrupter resistance” AND “preschool child^*^”. Full search strategy can be seen in Supplementary Table S1. Reviews, editorials, case reports and conference proceedings were excluded. Any papers including only children ≥ 6 years were excluded. Abstracts of identified documents were reviewed and screened by SC, with second author DG assisting with inclusion queries. All included papers were reviewed by SC.

## Results

A total of 626 papers were screened of which 61 were eligible for inclusion, Supplementary Figure S1. They included papers from 4 regions: 30 (49.2%) from Asia, 17 (27.9%) from South America, 9 (14.7%) from Africa and 5 (8.2%) from Europe and the Middle East. Five lung function tests were most commonly reported: 40 (65.6%) spirometry, 7 (11.5%) FeNO, 15 (24.6%) oscillometry, 2 (3.3%) interrupter technique and 2 (3.3%) tidal breathing measurements. There were no papers including MBW. The papers covered test feasibility (19, 31.1%), clinical applications (46, 75.4%) and epidemiological studies (13, 21.3%). Clinical studies focused on development of reference tools (19/46, 41.3%). and on specific diseases: asthma (15/46, 32.6%), of which 4 papers in addition to asthma included allergic rhinitis, air pollution, obesity and allergic bronchopulmonary aspergillosis (ABPA); CF (5/46, 10.9%); 1 bronchiolitis obliterans, 1 recurrent wheeze, 1 systemic sclerosis. Epidemiological studies assessed the impact of air pollution (10/13, 77%); electronic waste (e-waste) (2/13, 15.4%) (27, 28) and the effect of antenatal omega 3 fatty acid supplementation (1/13, 7.7%) on lung function. E-waste is accumulated discarded or broken electronic devices which is becoming the largest amount of waste in the world (27, 28). As spirometry, FeNO, oscillometry or tidal breathing were most commonly used they were the focus of this review.

### Spirometry

The majority (40, 68%) of papers included spirometry, (Table 1). Spirometry was used to develop reference equations, diagnose and manage respiratory diseases including asthma or CF, assess the impact of air pollution, electronic exposures (e-exposures) or socio-economic status on lung function. Twenty (50%) studies were from Asia, 9 (22.5%) from South America, 7 (17.5%). from Africa and 4 (10%) from Europe and the Middle East.

**Table 1:** Details of included studies using spirometry (*n* = 40).

**Authors, Year**	**Country**	**Age**	**No. of patients**	**Study type**	**Theme**	**Main finding**
Zhu et al. (63)	China	5–12yr; little group-divided into groups 5–7 years	121		asthma, allergic rhinitis	Spirometry was used to assess factors associated with FeNO. A greater peak expiratory flow in addition to a greater age, height/weight and level of total IgE are related to higher FeNO levels
He et al. (49)	China	5–13 years	43		air pollution	No significant associations were noted between personal PM_2.5_ exposure and spirometry.
Kang et al. (56)	China	4–12 years	286-asthma, 301-control		asthma	A BDR threshold of ≥ 7.5% may be more valuable compared to ≥ 12% in childhood asthma
Leung et al. (55)	China	2–7 years	1341		asthma	The minor allele SNP (rs408223), of CDHR3 was associated with lower FEV_0.5_ (β = −2.411, P = 0.004), and FEV_0.5_/FVC (β = −1.292, *P =* 0.015)
Sun et al. (52)	China	3–10 years	112	experimental study	asthma	Pulmonary function indices (FVC, FEV_1_ and PEF), were significantly higher (*p* < 0.001), in the observational group (treated with montelukast and budesonide), than the control group (budesonide alone), Treating cough variant asthma with montelukast combined with budesonide is more effective than budesonide alone.
Wang et al. (58)	China	2–5 years	120	randomized, double-blind placebo-controlled trial	acute asthma	Treating acute asthma exacerbation with montelukast compared to placebo demonstrated no significant difference in the PEF and FeV_1_.
Zeng et al. (28)	China	5–7 years	206		e-waste exposure	Taken together, birth weight and chest circumference may be good predictors for lung function levels in preschool children
Zeng et al. (27)	China	5–7 years	206		e-waste exposure	Children living in the exposed area have lower lung function (FVC and FEV_1_). Levels compared to unexposed children. Haemoglobin levels may be a good predictor for lung function- one unit of haemoglobin (1 g/L). Decline was associated with 5 mL decrease in FVC and 4 mL decrease in FEV_1_
Jian et.al. (35)	China	4– 80 years	7115	cross-sectional study	reference equation	This study established new reference values for the Chinese population 4 to 80 years. The “South East Asian” and “North East Asian” GLI reference equations under or overestimated the FEV_1_, FVC, and FEV_1_/FVC. Local Chinese equations underestimated FVC and FEV_1_
Sonnappa et al. (44)	India and UK	5–12 years	1039	prospective cross-sectional study	socio-economic circumstance	Spirometry differences were assessed between children from urban, semiurban, and rural schools. There were significant reductions in FEV1 and FVC in Indian-semiurban and Indian-rural children when compared with Indian-urban children.
Kumari et al. (57)	India	5–15 years	106	cross-sectional	asthma, ABPA	Percentage predicted values of FEV_1_ and FEF_25−75_ were lower in asthmatic children with allergic bronchopulmonary aspergillosis (ABPA). Compared to no ABPA, but this did not reach statistical significance. PEF that was significantly higher in children with aspergillus sensitization (AS). Compared to those without AS (*P=* 0.046).
Kumar et al. (50)	India	5–18 years	620	cross-sectional study	asthma, obesity	Obese children with asthma (Group 1). Had significantly lower lung function compared to non-obese asthmatic children(Group 2). FEV_1_,
						FVC, FEF_25−75%_, PEF for Group-1 were 66.3 ± 9.9,63.5 ± 4.2,54.2 ± 5.7,67.4 ± 8.4. FEV1, FVC, FEF_25−75%_, PEF for Group-2 were 74.07 ± 3.5,77.4 ± 7.2, 60.1 ± 2.1, 71.6 ± 2.4. P values were <0.001, <0.001, <0.001, <0.05 respectively.
Gulla et al. (92)	India	138–120 months	46	retrospective control study	cystic fibrosis	Children with viral infection (Group I). Had adverse outcome in form of greater worsening of Shwachman clinical scores, number of pulmonary exacerbations requiring antibiotic usage, need for intravenous antibiotics, hospitalization rates and mortality. Spirometry decreased in both groups decrease in lung function in both groups but was not significant
Bolla et al. (37)	India	5–15 years	790	cross-sectional study	reference equation	Separate equations in males and females were generated with age, weight and height as predictors. No comparison to other reference equations were made.
Lum et al. (42)	India and UK	5–17 years	8124	observational	reference equation	“GLI-Black” equations were most useful for interpreting South-Asian data and “GLI-Other” for North Indian data. When using GLI-predicted values from White Europeans, FEV_1_ and FVC in South-Asian children were approximately 15% lower. There was an association between socio-economic circumstances (SEC), and lung function. Lung volumes were significantly lower in those living in rural areas or exposed to poorer SEC.
Asrul et al. (45)	Malaysia	5 and 6 years	120	cross-sectional comparative study	air pollution	There was a significant difference in indoor air quality between urban and suburban preschools. FVC and FEV_1_ among urban children were significantly lower compared to the suburban children. Exposures to indoor air pollutants, especially PM_2.5_ increases the risk of getting lung function abnormalities.
Choo et al. (46)	Malaysia	4–6 years	630	cross-sectional comparative study	air pollution	Urban area preschools have higher CO, PM_10_ and PM_2.5_ concentration compared to from suburban and rural areas. FVC, FEV_1_, FVC% predicted and FEV_1_% predicted values were significantly lower among children from urban and suburban area preschools compared to rural preschools.
Kamaruddin et al. (93)	Malaysia	5–6 years	100	cross-sectional comparative study	air pollution	Significant associations between PM10 and VOCs with FEV_1_% were noted (PR = 5.55, 95% CI = 2.189–14.07), (PR = 6.15, 95% CI = 2.565–14.73), respectively in exposed compared to unexposed children.
Rawi et al. (47)	Malaysia	5–6 years	11	cross sectional study	air pollution	Studied preschools had a significantly higher PM and CO concentration compared to the comparative preschools. FVC, FEV_1_, FVC% and FEV_1_% predicted values were significantly lower among studied group.
Asif et al. (39)	Pakistan	5–14 years	3275	cross-sectional study	reference equation	Reference range equations were developed with predictors that included age, height, and weight. Separate equations for males and females were generated. No comparisons made to other studies.
Ventura et al. (59)	Brazil	1–15 years(median age 3.75 years)	38 with CF,31 control	longitudinal study	cystic fibrosis	Pasclerosisrticipants with higher C-reactive protein/albumin ratio at the baseline had higher odds of FEV1 ≤ 70% after three years of follow-up.
Veras et al. (31)	Brazil	6 years and younger	74	cross-sectional descriptive	feasibility	The spirometry success rate was 82%. Performance improved with age.
França et al. (32)	Brazil	4–6 years	195		reference equation	Reference range generated using height as a predictor. One equation for males and females. No comparison to GLI 2012
Jones et al. (34)	Brazil	3–12 years	1990	cross-sectional observational study	reference equation	Equation generated significantly from those currently in use in Brazil-Underestimate FVC and FEV1 values.
Burity et al. (33)	Brazil	3–6 years	425	prospective study	reference equation	Full expiratory curves are more difficult to obtain in preschool children. In addition to height, gender also influenced the measures of FVC and FEV_1_
Matos et al. (53)	Brazil	4–12 years	1129	cross-sectional study	asthma	Overweight children have less respiratory capacity, and was associated with lower FEV_1_/FVC ratios (PR =1.37; 95% CI 1.14, 1.64)
França et al. (30)	Brazil	4–6 years	47		asthma	83% success rate for performing spirometry
Ardura-Garcia et al. (64)	Ecuador	5–15 years	264	cohort study	asthma	Spirometry did not predict asthma recurrence.
Heinzerling et al. (48)	Guatemala	5–8 years	506	prospective cohort study	air pollution	A significant decrease in PEF [173 mL/min/year (95% CI −341 to −7)], and a non-significant decrease in FEV1 growth were observed with later stove installation at 18 months compared with stove installation at birth
Bougrida et al. (38)	Algeria	5–16 years	208		reference equation	Several predictors in the reference range and these include height, weight, age, gender BSA, BMI. Separate equations for males and females. There were significant differences in FeV_1_ between the measured and predicted values from published reference equations except for a USA reference equation.
Jiffri et al. (54)	Egypt	1–15 years	120 asthma,120 controls		asthma	There is an association between the TNFA −308G>A polymorphism and susceptibility to asthma. Spirometry used to classify patients into asthma severity namely mild intermittent asthma, mild persistent asthma, moderate persistent asthma, or severe persistent asthma
Akodui et al. (40)	Nigeria	5–12 years	100	cross-sectional study	sickle cell anaemia, reference equation	Preferred proxy for spirometry indices in children with sickle cell anaemia may be arm span
Thacher et al. (94)	Nigeria	5–11 years	299	cross-sectional study	asthma, air pollution	The relationship between smoke exposure and airway obstruction in households that did and did not use firewood daily was not significant (mean FEV1/FEV6 of 0.95 and 0.97, respectively; *P* = 0.41). There was a significant decline in predicted FEV_1_ with age (*p* < 0.001)
Corten et al. (95)	South Africa	5–8 years	12	cross-sectional study	cystic fibrosis	There were significant correlations between PEF and manual dexterity and between FVC % predicted and balance scores Poorer lung function may affect motor development.
Smith et al. (43)	South Africa	5–95 years	4223	cross-sectional population-based study	reference equation	GLI2012 “Other” had the best fit for Black African individuals and Mixed Ethnicity group when using z-scores. The Caucasian individuals demonstrated a good fit with the GLI2012 “Caucasian” equation and participants of Asian ancestry demonstrated a good fit to the “Southeast Asian” and “Black” equation.
Sibanda et al. (96)	Zimbabwe	1–94 years	240 (49 between 1- 16 years of age)	observational study	systemic sclerosis	The mean FEV_1_/FVC ratio for all the patients combined was Significantly higher than predicted for age, gender, ethnicity, and BMI
						suggesting a restrictive pattern. The severity of the restrictive changes varied with the types of autoantibodies detected.
Ghasempour, M. et al. (51)	Iran	5–15 years	73	description-observation	asthma	The average FEV_1_/FVC parameter in the cough variant asthma group was 89.44 ± 13.07, and 72.35 ± 8.47 in the classic asthma group, with a significant difference between the two groups (*p* < 0.05). Patients with cough variant asthma FEF_25−75%_ were lower than expected. Spirometry can be used in the diagnosis of cough variant asthma.
Tabatabaie et al. (36)	Iran	4–10 years	495		reference equation	Reference range equations were generated for both males and females using height and age as predictors. When compared to previous published international equations significant differences were noted.
Al-Qerem et al. (29)	Jordan	3–5 years	765	random sampling	reference equation	The GLI 2012 for Caucasians is a reasonable fit for Jordanian preschool aged children.
Sasihuseyinoglu et al. (60)	Turkey	mean age7.83 years	80	retrospective study	cystic fibrosis	There were significant correlations between the Bhalla score and FEV_1_, FVC, and FEF_25−75%_

*PR, Prevalence Ratio; CI :Confidence Interval; PM, particulate matter; PM_2.5_ :particulate matter 2.5; PM_10_, particulate matter 10; PEF, peak expiratory flow; SNP, single-nucleotide polymorphism; FEV0.5, forced expiratory volume in 0.5-second; FVC, forced vital capacity*.

Success rates for spirometry in preschool children increased with age (29). Children between 4–6 years of age achieved a success rate between 82–85%, (30–32). while children between 3–5 years and 3–6 years of age, success rates were 68.4% and 42% respectively (29, 33).

A number of reference ranges were generated for individual population groups in LMIC, which were compared to either local reference ranges and/or The Global Lung Function Initiative (GLI) 2012 equation (32–39). Many resulted in an over estimation/underestimation of lung function for the population assessed, highlighting the complexity of population differences in lung function. A Nigerian study determined reference equations for children with sickle cell anemia (40). Published international equations also used arm span to determine height for spirometry (41). The GLI 2012 “Caucasian” provided a reasonable fit for Jordanian children (29). When assessing spirometry in two groups of healthy children with Indian ancestry, one living in UK and the other in India, the GLI equations for best fit differed: the “GLI-Black” equation was most useful for interpreting the South-Asian data and “GLI-Other” for North Indian data (42). Similarly in a South Africa population the GLI 2012 “GLI-Caucasian” provided a good fit for the Caucasian population, ”GLI-Black “ and “GLI-Southeast Asian” was a good fit for the Indian population and “GLI-Other” fit the Black African and Mixed ancestry populations well (43). These findings highlight the importance of multiple factors, including environment and socioeconomic exposures that impact population differences (42, 43).

Numerous environmental factors were reported to impact on lung function. Children living in rural areas or exposed to poorer socio-economic circumstances had lower lung function compared to those in urban areas (42, 44). Exposures to volatile organic compounds, particulate matter 10 (PM_10_) or carbon monoxide (CO) were associated with a decrease in FEV_1_ and FVC in exposed preschool children (45–47).

In a study in Guatemala, where CO was used as a proxy for PM_2.5_, timing of chimney stove installation was compared to cooking over open fires, and showed a decrease in PEF of 173 ml/min/year (95% CI −341 to −7). with chimney stove installation at 18 months compared to installation at birth (48). A Chinese study failed to demonstrate any significant association between PM_2.5_ exposure and any of the spirometry lung function measures, however an increase in oscillometry resistance was noted suggesting that oscillometry may be a more sensitive measure (49). In addition to air pollution, e-waste is a growing concern. Chinese children living in e-waste exposed areas had significantly lower birth weight, chest circumference and spirometry lung function compared to those in unexposed areas (27, 28).

The majority of included papers used spirometry in the clinical diagnosis and management of pediatric obstructive lung disease including asthma and CF. The studies investigated genetic predisposition to asthma, and management of acute, poorly controlled and cough-variant asthma (CVA). and the impact of obesity (50–53). Genes associated with increased susceptibility to asthma and lower spirometry indices were identified in Chinese and Egyptian children (54, 55). One study assessed current definitions of BDR, suggesting that a BDR of >7.5% may be more valuable in young children rather than the adult defined 12% (56). Another study included assessing impact of ABPA on spirometry of asthmatic children (57). In a Chinese study acute asthma did not respond to adding montelukast to the regular regimen (58), however it was noted that in children with CVA, cough associated with chronic airway allergic inflammation without wheeze, had a significant improvement in FEV_1_, FVC, and PEF (*p* <0.001). with montelukast and budesonide compared to budesonide alone, while another study noted that FEV_1_/FVC was normal in CVA compared to patients with asthma (*p* <0.001) (51, 52).

Spirometry was also used in South American, Turkish and South African children to monitor lung function in cystic fibrosis (59), and showed good correlation between CT scan Bhalla score and FEV_1_, FVC, FEF_25−75_ (60).

### Fractional Exhaled Nitric Oxide

Of the 7 papers using FeNO, 4 (57%) clinical studies measured FeNO to assess risk and treatment response of asthma or recurrent wheeze; and 3 epidemiological studies assessed the impact of IAP on airway inflammation in children. These included studies mainly from Asia (5 from China, 1 from Thailand) and 1 from Ecuador, South America. There were no published preschool studies from Africa. Included studies are summarized in Table 2.

**Table 2:** Detail of included studies that assessed fractional exhaled nitric oxide (*n* = 7), oscillometry (*n* = 15), interrupter technique (*n* = 2). and tidal breathing (*n* = 2).

**Authors, Year**	**Country**	**Age**	**No. of patients**	**Study type**	**Theme**	**Main finding**
**Fractional exhaled nitric oxide**						
Zhang et.al (62)	China	5 years	507	cross-sectional study	air pollution	Indoor and outdoor PM2.5 levels in day care centres were associated with higher levels of FeNO. FeNO levels were also associated with current wheeze and physician diagnosed pneumonia.
Han et.al. (65)	China	4–11 years (4–6 and 7-11 years)	142	cross-sectional descriptive study	asthma	Family management (FM), describes how family members cooperate and integrate the management of childhood chronic disease into their daily family life. FM was closely related to asthma control and could significantly predict FeNO value and C- ACT score.
Li et al. (22)	China	32–48.7 months	88		recurrent wheeze	sRAGE may be a novel biomarker of inflammation of the respiratory tract. There was a significant negative correlation between serum sRAGE and FeNO (*p* < 0.001). In the high-risk asthma group, sRAGE levels increased significantly while FeNO decreased significantly after Pulmicort therapy
Zhu et al. (63).	China	5–12 yr;little group classified as 5–7 years.	121		asthma, allergic rhinitis	Height and total IgE are well correlated with FeNO in asthmatic children greater age, height/weight, peak expiratory flow (PEF), and higher level of total IgE (*p* < 0.001) are associated with higher FeNO levels
He et al. (49)	China	5–13 years	43		air pollution	An increase in 24-h personal PM_2.5_ exposure one day prior to the clinic visit was associated with a significant increase in of FeNO (airway inflammation), of 9.6%
Siwarom et.al (61)	Thailand	29–72 months	436	randomised control study	air pollution	The mean FeNO levels were statistically different in each season (*p* < 0.001). FENO levels had a strong association with high benzene levels (OR 5.9; 95%CI 1.5–22.9; *p*-value = 0.01).
Ardura-Garcia, C.et al. (64)	Ecuador	5–15 years	264	cohort study	asthma	FeNO level did not predict asthma recurrence.
**Oscillometry**						
Zhang et.al. (78)	China	3–14 years	120	retrospective study	upper airway obstruction	R_5_ in the OSAHS group was significantly higher than that in the non-OSAHS group (*P =* 0.0025)
He et al. (49)	China	5–13 years	43		air pollution	An increase in 24-h personal PM_2.5_ exposure one day prior to the clinic visits was associated with a significant increase in total airway resistance (R_5_) of 6.3%, small airway resistance (R_5−_R_20_) of 15.8%
Li et al. (82)	China	<14 years	42	case review	bronchiolitis obliterans	In children with bronchiolitis obliterans impulse oscillometry showed an increase in Z_5_ (147.5 ± 19.3% of the predicted value, normal: less than 120% of the predicted value), R_5_ (140.4 ±12.8% of the predicted value, normal: less than 120% of the predicted value), and X_5_ (226.5 ± 13.4% of the predicted value, normal: less than 120% of the predicted value). This suggesting increased peripheral airway resistance.
Udomittipong et al. (70)	Thailand	3–7 years	291	cross-sectional study	reference equation	Reference values for respiratory impedance using FOT were generated using height and arm span were generated.
Udomittipong et al. (73)	Thailand	3–6 years	150		asthma	Cut-off values for evaluating bronchodilator response in FOT were determined: Rrs6: −23%, Rrs8: −20%, Rrs10: −20%, Xrs6: 36%, Xrs8: 60%, and Xrs10: 43%.
Gupta et al. (69)	North India	2–18 years	345	prospective interventional study	asthma	Oscillometry is a useful tool to assess lung function and airway reversibility in asthmatic children. It can provide an objective measurement in children unable to perform spirometry.
Medeiros et.al. (74)	Brazil	3–6 years	76	cross -sectional study	respiratory symptoms	IOS in children with respiratory symptoms were higher pre-bronchodilator for R_5_ Hz and R_5−20_Hz compared to those children without respiratory symptoms.
Duenas-Meza et al. (77)	Colombia	3–5 years	96	cross-sectional study	reference equation	Normal IOS reference range equations were determined, and height was the only predictor. A fall in R_5_Hz of 28% or an increase in X_5_Hz of 36% postbronchodilator can be considered as an upper limit of normal.
Gutiérrez-Delgado et.al. (68)	Mexico	birth cohort until 5 years	772	double blind, randomized, placebo-controlled study	Intervention with DHA	Prenatal DHA supplementation did not influence IOS values with respect to resistance and reactance at 6, 8, and 10 Hz
Gochicoa-Rangel et al. (71)	Mexico	2.7–15.4 years	283		reference equation	Reference range equations were derived for impulse oscillometry. Predictors include age and height. Marked differences were noted between the derived reference equation when compared to other studies. Separate reference ranges for male and females were generated.
Gochicoa-Rangel et.al. (75)	Mexico	4–15 years(mean age 8.6)	224	cross-sectional study	reference equation	Due to the robust adjustment of the equation derived from Gochicoa-Rangel et al. (71) this equation has been recommended for clinical and research purposes in a Mexican population.
Shackleton et al. (72)	Mexico	3–5.2 years	584	double-blind, randomised, placebo-controlled clinical trial	reference equation	Reference ranges for FOT were generated for Mexican children. Height was the only predictive factor and the same equation for males and females was used. An Australian reference range equation overestimate lung function in Mexican children.
Dubowski et.al. (67)	Ghana	4 years	112	prospective study	infection exposure	Infants exposed to a less diverse NPM (nasopharyngeal microbiota) had a higher small airway resistance (R5–R20 = 17.9%, 95% CI 35.6, 0.23; *p* = 0.047). Compared to a more diverse phenotype
Dutta et.al. (66)	Nigeria	2 years (mean age 2.9 years)	223	randomised control trial	air pollution	Increase in postnatal household air pollution (PM_2.5_). were significantly associated with higher airway reactance at 5 Hz (X5 Hz; *P=* 0.04)
Er et al. (76)	Turkey	3–7 years	151		reference equation	Reference values for IOS in healthy Turkish children were determined. Resistance was significantly correlated with height and reactance was significantly correlated with age (p <0.05–). Separate equations were derived for males and females.
**Interrupter technique**						
Rocha et al. (79)	Brazil	5 to 18 years (mean 10,79 years)	38	cross-sectional study	cystic fibrosis	Interrupter resistance (Rint). correlates well with spirometry. There was a strong correlation between inverse Rint and FEV1 (r = 0.8; *p* <0.001), and moderate correlation between inverse Rint and FEF25–75% (r = 0.74; *p* <0.001). Rint was not accurate in evaluating bronchodilator response.
Gochicoa et.al. (80)	Mexico	24 days to 6.6 years	264	prospective, cross-sectional descriptive study	reference equation	Reference values for interrupter technique (Rint), was determined in Mexican children. There was an inverse relationship between Rint and height. Females had a higher Rint than males (*P* = 0.054).
**Tidal breathing**						
Kumar et.al. (81)	India	3 years	310	prospective birth cohort study	acute respiratory infections	The ratio of tidal expiratory flow (TEF) at 25 or 50% of tidal expiratory volume to peak TEF (TEF50 or TEF25/peak TEF) at 3 years was significantly increased in children who had an acute respiratory infection in infancy.
Li et al. (82)	China	<14 years	42	case review	bronchiolitis obliterans	The tidal breathing analysis revealed a decreased tPTEF% tE (18.2 ± 0.26%, normal: more than 40%), or VPEF%VE (21.7 ± 0.32%, normal: more than 40%).

*FeNO, fractional exhaled nitric oxide; OR, odds ratio; CI, 95% confidence interval;PM_2.5_, particulate matter 2.5; C-ACT, Childhood Asthma Control Test; sRAGE, Receptor for Advanced Glycation End products; Z_5_, magnitude of respiratory impedance; R_5_ :total respiratory resistance; X_5_:distal capacitive reactance; obstructive sleep apnea hypopnea syndrome (OSAHS); R5Hz, total resistance at 5 Hz; R20 Hz :central resistance at 20 Hz; R_5−_R_20_ Hz, difference between resistance at 5Hz and 20 Hz; IOS, Impulse oscillometry; PM2.5:particulate matter 2.5; CI:95% confidence interval; Rrs6, Rrs8, Rrs10, Resistance at 6 Hz, 8Hz and 10Hz; Xrs6, Xrs8, and Xrs10, Reactance at 6 Hz, 8 Hz and 10Hz; DHA, docosahexaenoic acid; tPTEF% tE, ratio of time to reach peak tidal expiratory flow to total expiratory time VPEF%VE, ratio of volume to reach peak expiratory flow to total expiratory volume*.

The success rate for performing FeNO in preschool children ranged between 86–99%. Thai children living in a metropolitan area attending day care, average age of 50.1 months (range 29–72 months), achieved a success rate between 86–93% with data collected over 3 time points (61). A cohort of 507 Chinese children aged 5 years achieved a success rate of 99% (62). Over half of studies (57%) used a Niox Mino analyzer (Aerocrine, Solna, Sweden). to measure FeNO using the single breath technique. Most studies used the recommended normal standards (26). No studies have explored population differences in FeNO.

All 3 studies assessing impact of air quality on respiratory health found that environmental pollution, including benzene and PM_2.5_, were associated with high FeNO (49, 61, 62).

Studies from China and one from South America, used FeNO measurement in the management of preschool asthma (63–65), noting the strong association with IgE mediated inflammation and ICS efficacy (63). However, it was also suggested that FeNO may be less useful in the preschool age group for detecting ICS response, as the mean FeNO level was significantly higher only in the “older” age group than the cut off values reported in other studies for the diagnosis of asthma (63). FeNO was not able to predict recurrence (64).

### Oscillometry and Interrupter Technique

Oscillometry has been successfully used in LMIC to assess the impact of early life exposures and for clinical management of children with respiratory disease. These include: 6 studies from Asia, 6 from South America, 2 from West Africa and 1 from the Middle East summarized in Table 2. The studies used a range of commercially available equipment including both impulse oscillometry (IOS). and airwave oscillometry (AOS).

Oscillometry has proven to be effective in assessing the impact of early life exposures on preschool lung function (49, 66). An increase in household PM_2.5_ exposure increased airway reactance in Nigerian children, similarly high personal exposure to PM_2.5_ was associated with an increase in small airway resistance (R_5−20_), total airway resistance (R_5_). and resistance frequency dependence (R_5−20_), representing small airway disease, in asthmatic Chinese children living outside Shanghai (49, 66).

In a Ghanaian longitudinal cohort, infants exposed to a less diverse nasopharyngeal microbiome had a higher small airway resistance compared to a more diverse microbiome at 4 years of age (67). Further, in a Mexican birth cohort study, prenatal omega 3 fatty acid supplementation in pregnancy did not influence preschool lung function at 36, 48 or 60 months (68).

Oscillometry was easily performed by preschool children with the overall success rate ranging between 74–98% in studies making it a particularly attractive option (69–73). As a clinical tool oscillometry for preschool children is supported by studies that are able to detect differences in prebronchodilator lung function of preschool children with respiratory symptoms compared to those without respiratory symptoms (74).

Reference data for Mexican, Thai, Turkish and Colombian children have been collected (70–72, 75–77), facilitating the use in diagnosis of respiratory disease. It is also a useful tool to assess airway reversibility in asthmatic children and cut off values for bronchodilator response were proposed (69, 73). A Chinese study in children with obstructive sleep apnea hypopnea syndrome (OSAHS). demonstrated an increase in total airway resistance in children with OSAHS compared to children with snoring but without OSAHS (78).

The interrupter technique was used in 2 South American studies, one of which looked at the development of reference values for newborn, infants and preschool children, while the other measured Rint in children with cystic fibrosis and found that Rint correlated well to spirometry FeV_1_ and FEF_25−75_, but not accurate in determining bronchodilator response (79, 80).

### Tidal Breathing

Tidal breathing was used in 2 studies, one an Indian birth cohort and the other a Chinese retrospective cohort study (81, 82), (Table 2). Tidal breathing measurements were in keeping with an obstructive pattern in patients with bronchiolitis obliterans (82). Indian children who had an acute respiratory tract infection in infancy had increased ratios of tidal expiratory flow (TEF) at 25 or 50% of tidal expiratory volume to peak TEF (TEF50 or TEF25/peak TEF) at 3 years suggesting increased airway resistance (81).

## Conclusion

Preschool lung function tests used in LMIC were feasible with high success rates. Success with spirometry increased with age (29), and oscillometry had higher feasibility compared to spirometry. All tests were useful for clinical application and epidemiological studies. Comprehensive preschool testing including spirometry, FeNO, oscillometry and tidal breathing were only reported from China, and only 19 countries of 137 registered LMIC (14%) were represented in the review, a large discrepancy as the majority (80%) of children live in LMIC, where the burden of early life respiratory disease and exposures known to cause illness are high. These include air pollution, maternal smoking, a high infectious load including tuberculosis, and high rates of preterm birth (2, 83–86). Lung functions tests assessing these vulnerable groups are lacking. A number of studies explored the effects of air pollution; and the studies suggest that air pollutants increase airway inflammation and may in part explain the association between household air pollution and recurrent wheeze (83). Given the burden of early life exposures and the need to identify preventative measure, priority should be given to strengthening access to lung function assessment tools.

The majority of clinical studies focussed on asthma diagnosis and management, including defining BDR in young children. The prevalence of asthma in LMIC is high, with a temporal increase in severe asthma (87). Of particular concern is that up to 40% of children in LMIC with severe symptoms are undiagnosed (87, 88). Oscillometry was useful, and more sensitive than spirometry, in measuring airway resistance and cut off values for BDR response was also determined. FeNO has been identified as a useful adjunct in diagnosis and informing treatment in asthmatic patients (89). Access to these tests to improve diagnosis and management is needed for the many children living in LMIC.

Other clinical uses have included management of chronic lung conditions like CF, bronchiolitis obliterans, OSAHS, obesity and systemic sclerosis all of which are associated with respiratory complications. Lung function tests assessing vulnerable groups such as children born preterm, those living with HIV or exposed in utero and those with a history of pulmonary TB and other early life lower respiratory tract infections are lacking in children from LMIC.

The importance of reference range equations were highlighted in these studies. A healthy standard is needed for lung function tests to distinguish between health and disease in different populations. The GLI reference equations attempt to address this, however unique environmental exposures including in-utero exposures influence lung development and this needs to be considered when developing reference equations (90). Research studies assessing impact of exposures require appropriate patient samples and statistical modeling and should include both exposed and unexposed control groups.

There are currently no published data for MBW in preschool children in LMIC settings, however data on infant testing have been published in South Africa (3, 91). There is limited data on tidal breathing and the interrupter technique. Impulse and airway oscillometry use different input signals and this may impact results especially at high impedance, which is associated with disease. Furthermore, FeNO single breath and tidal breathing measures have different interpretation of normal ranges, and the results are not interchangeable. These factors may affect the interpretation and comparison of data.

Challenges to accessing lung function testing need to be addressed, these include lack of trained personnel as each lung function test requires specialized training skills and lack of financial resources to support development and implementation of these tests. Spirometry and oscillometry are relatively inexpensive, whereas FeNO and MBW are currently more expensive further limiting access. Equipment maintenance and poor access locally to consumables and technical support incur further costs. This reduces research outputs and lack of robust data to better diagnose, prevent and manage respiratory disease in these settings (5).

In conclusion, preschool testing in LMIC is feasible, both epidemiologically and clinically. It has the potential to be useful in strengthening the diagnosis, management and prevention of respiratory diseases in younger children but is underutilized. Spirometry still remains a key clinical and epidemiological tool in LMIC, however has limitations especially in young children. Understanding and addressing the challenges for improving access to these tools is needed in order to strengthen the prevention, early diagnosis and management of childhood respiratory disease in LMIC.

## Author Contributions

SC, DG, and HZ conceived the idea. SC and DG reviewed the literature and drafted the manuscript. HZ provided further input into the manuscript. All authors reviewed, contributed, and approved the final manuscript.

## Funding

DG was funded by the Wellcome Trust (204755/z/16/z). HZ was funded by the SA-Medical Research Council.

## Conflict of Interest

The authors declare that the research was conducted in the absence of any commercial or financial relationships that could be construed as a potential conflict of interest.

## Publisher's Note

All claims expressed in this article are solely those of the authors and do not necessarily represent those of their affiliated organizations, or those of the publisher, the editors and the reviewers. Any product that may be evaluated in this article, or claim that may be made by its manufacturer, is not guaranteed or endorsed by the publisher.
