# Supplementary material for: Lung Function in Preschool Children in Low and Middle Income Countries: An Under-Represented Potential Tool to Strengthen Child Health
Source: Front Pediatr. 2022 Jun 6;10:908607. doi: 10.3389/fped.2022.908607 (PMC9234953; doi:10.3389/fped.2022.908607)
Supplement: Supplementary file 1 [file Table_1.docx]

Supplementary table and figure

Table S1: Search strategy

| **Search** | **Database** |  |
| --- | --- | --- |
| “Respiratory Function Test*” OR “Lung function” OR “Lung function test*” OR “pulmonary function” OR “pulmonary function test*” OR “respiratory function” OR “respiratory function test*” OR “Respiratory outcome*” OR “pulmonary outcome*” OR “lung outcome*” OR “multiple-breath washout” OR “multiple breath washout” OR MBW OR “multiple-breath nitrogen washout” OR “multiple breath nitrogen washout” OR MBNW OR FOT OR “Forced oscillation technique” OR” tidal breathing” OR “fractional excretion of nitric oxide” OR Feno OR Spirometry OR Oscillometry OR “impulse oscillometry” ” OR “interrupter technique” OR “interrupter resistance” AND" preschool child*" OR "pre-school child*" | ***Scopus-EMBASE** | **Total papers**: 355 |
|  | ***** **EBSCO host** | **Total papers** :41  - CINAHL:6  - Africa-Wide Information :2  - Health Source: Nursing/Academic  Edition:33 |
|  | ***Web of science** | **Total papers** :44  -core collection:40  -Sielo:4 |
| "Respiratory Function Tests" OR Lung function OR Lung function test OR Lung function tests OR pulmonary function OR pulmonary function test OR pulmonary function tests OR respiratory function OR respiratory function test OR respiratory function tests OR Respiratory outcome OR respiratory outcomes OR pulmonary outcome OR pulmonary outcomes OR lung outcome OR lung outcomes OR multiple-breath washout OR multiple breath washout OR MBW OR multiple-breath nitrogen washout OR multiple breath nitrogen washout OR MBNW OR oscillometry OR FOT OR Forced oscillation technique OR tidal breathing OR “fractional excretion of nitric oxide” OR Feno OR spiro OR spirometry OR "Spirometry" OR "Oscillometry" OR “impulse oscillometry ” OR “interrupter technique” OR “interrupter resistance” AND "preschool child" OR "preschool children" OR "pre-school child" OR "pre-school children" OR "child, preschool" | ***** **PubMed** | **Total papers** :186 |

*Limits to 10 years

Records screened after duplicates removed (n=431)

Records identified through database (n=626) jj((n=485searches

Identification

Screening

Eligibility

Full test articles assessed for eligibility (n=61)

Duplicates removed (n=195)

**Reasons for exclusion (n=370)**

Adult outcome-17

Adult and child not differentiated-4

Review/case report/conference proceeding-15

Guidelines-1

Insufficient information-14

Not a LMIC-18

Not in English-9

Other PFT-22

Out of selected age range-130

PFT not an outcome-132

Protocol-4

Retracted-1

Editorial-3

Included

Numbers included (n=61)

Figure 1: Flow diagram of final papers included
